# Small Peptides in the Detection of Mycotoxins and Their Potential Applications in Mycotoxin Removal

**DOI:** 10.3390/toxins14110795

**Published:** 2022-11-16

**Authors:** Zitong Zhao, Zhenzhen Zhang, Haoxiang Zhang, Zhihong Liang

**Affiliations:** 1 College of Food Science and Nutritional Engineering, China Agricultural University, Beijing 100083, China; 2 The Supervision, Inspection and Testing Center of Genetically Modified Organisms, Ministry of Agriculture, Beijing 100083, China; 3 Beijing Laboratory for Food Quality and Safety, College of Food Science and Nutritional Engineering, China Agricultural University, Beijing 100083, China

**Keywords:** small peptides, artificial enzymes, mycotoxin detection, mycotoxin removal, mycotoxin control

## Abstract

Mycotoxins pose significant risks to humans and livestock. In addition, contaminated food- and feedstuffs can only be discarded, leading to increased economic losses and potential ecological pollution. Mycotoxin removal and real-time toxin level monitoring are effective approaches to solve this problem. As a hot research hotspot, small peptides derived from phage display peptide libraries, combinatorial peptide libraries, and rational design approaches can act as coating antigens, competitive antigens, and anti-immune complexes in immunoassays for the detection of mycotoxins. Furthermore, as a potential approach to mycotoxin degradation, small peptides can mimic the natural enzyme catalytic site to construct artificial enzymes containing oxidoreductases, hydrolase, and lyase activities. In summary, with the advantages of mature synthesis protocols, diverse structures, and excellent biocompatibility, also sharing their chemical structure with natural proteins, small peptides are widely used for mycotoxin detection and artificial enzyme construction, which have promising applications in mycotoxin degradation. This paper mainly reviews the advances of small peptides in the detection of mycotoxins, the construction of peptide-based artificial enzymes, and their potential applications in mycotoxin control.

## 1. Introduction

Mycotoxins are the secondary metabolites produced by fungi during growth and have been a global concern for a long period. Many fungal species usually produce them as a defense against the changing environment, which mainly including *Penicillium*, *Aspergillus*, and *Fusarium* [1]. The most frequently encountered mycotoxins in agricultural products and foods include aflatoxins (AFs), ochratoxin A (OTA), deoxynivalenol (DON), zearalenone (ZEN or ZEA), fumonisins (FBs), T-2 toxin (T-2), HT-2 toxin (HT-2), ergot alkaloids (EAs), citrinin (CIT or CTN), and patulin (PAT) [2,3]. Because of their highly resistive characteristics, mycotoxins tend to remain in the food chain and affect a broad range of agricultural products and foods, including wheat, rice, maize, fruit, and other related processed foodstuffs [4]. Mycotoxins can impose acute or chronic effects on both humans and animals, leading to impaired immunity, endocrine abnormalities, and carcinogenic effects [5]. Many tend to co-occur with other sometimes structurally unrelated mycotoxins, which may bring multiplicative and synergistic effects to human and animal health [6,7]. Therefore, maximum levels (MLs) for mycotoxins have been set in agricultural products and foods by various countries and organizations (Table 1).

Traditionally, analytical methods for mycotoxin detection can be divided into two categories: sophisticated instruments-based techniques for quantitative analysis and immunological techniques for the rapid real-time detection of bulk samples [8]. The instrumental analyses include high-performance liquid chromatography (HPLC), ultra-high liquid chromatography (UHPLC), and gas chromatography (GC) coupled with ultraviolet (UV), fluorescence (FLD), or mass spectrometric (MS) detection, allowing the largest range of mycotoxins to be determined with the highest sensitivity. However, these methods are only suitable for the analysis of mycotoxins in the laboratory not in real-time monitoring because of their expensive price, complicated sample preparation, time-consuming process, susceptible matrix interference, and the requirements of highly skilled analysts, which limit their wide application in resource-constrained regions. So, rapid methods that require only minimal sample preparation have been developed [9]. Most methods for rapid detection of mycotoxins are based on immunoassays [10]. Despite the possibility of expressing false-positive results or poor repeatability, the immunological method has the advantages of high specificity, high sensitivity, simple operation, and real-time detection, which make up for the above-mentioned shortcomings.

The enzyme-linked immunosorbent assay (ELISA) is the most frequently used technology for the rapid detection of mycotoxins [11]. The basic principle of the immunological assay is the reaction of antigens and antibodies. Therefore, it is important to prepare monoclonal antibodies that can specifically bind to small-molecule toxins in the ELISA method. As mycotoxins are small molecular haptens, mycotoxin-specific antigen preparation processes are complex, time-consuming, and labor-intensive, as well as having poor stability and high cost, which limits its wider application. Hence, chemically synthesized mycotoxin conjugates are inevitably introduced as coating or competitive antigens during the detection process. However, there are various drawbacks in the synthesis of mycotoxin conjugates, such as complex synthesis procedures, easy production of by-products, and the need to use poisonous mycotoxin standards that pose a threat to the safety of the detector and the environment. Thus, replacing mycotoxin conjugates with safe and renewable alternatives is essential for the development of eco-friendly immunoassays.

Small peptides can serve as competitors, inhibitors, mimotopes, drugs, reagents for affinity purification systems, and active elements in biosensors [12]. Recently, researchers found small peptides obtained from phages display peptide libraries can be used as biosensors for the detection of mycotoxins, and some of them are even more effective than antibodies [11]. Moreover, with the development of rational design technology, small peptides capable of having affinity to specific mycotoxins can be easily obtained by computer-aided design, screening, and solid-phase peptide synthesis [13]. Meanwhile, molecular modeling and simulation (MMS) methodologies capable of evaluating the performance of rationally designed peptide biosensors, with the potential to minimize biosensor development costs, improve product lifetime, and promote the multi-analyte detection of mycotoxins, are being developed [14]. According to the current reports, small peptides can act as coating antigens, competitive antigens, and anti-immune complexes in the detection of mycotoxins using immunoassays.

Although there are various methods to detect and even monitor mycotoxins in real time, contamination is still inevitable and remains a global problem [15]. Enzymes with high specificity, regioselectivity, and stereoselectivity can efficiently and specifically degrade specific mycotoxins such as AFB_1_, OTA, DON, and ZEN, and are becoming the most promising approach to address mycotoxin contamination after physical adsorption, chemical decomposition, and microbial detoxification [1,16]. However, few mycotoxin-degrading enzymes are applied commercially, and most enzymes are still being researched in the laboratory, because enzyme catalysis requires suitable conditions, usually effective under mild physiological conditions, but poorly tolerant to temperature, pH, and organic solvents, and easily losing catalytic activity. Therefore, it remains a challenging and meaningful mission to design a catalyst that can adapt to various complex and extreme industrial production environments, and also meet the requirements of catalyzing specific reactions. In recent years, with the research on the structure and catalytic mechanism of natural enzymes, artificial enzymes based on small peptides have become a research hot topic because of their promising break through the poor stability drawbacks of natural enzymes [17,18,19,20]. Despite the few relevant reports that are currently available [21,22], peptide-based artificial enzymes may provide a potential new approach for the removal of mycotoxins in the future.

**Figure 1:**
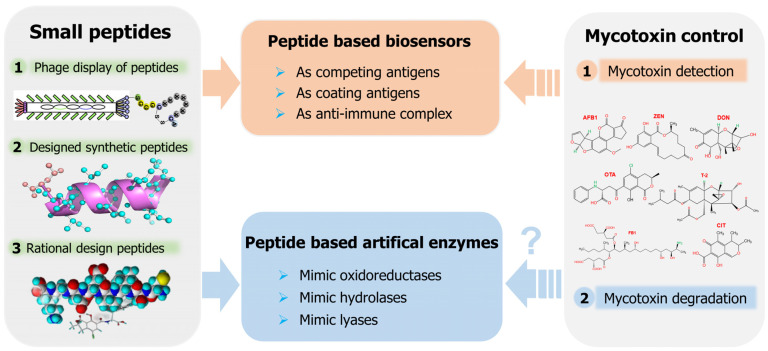
Small peptides in mycotoxin detection and potential applications in mycotoxin degradation.

Mycotoxin contamination not only harms human and animal health, but contaminated food and foodstuffs can only be discarded, which causes great economic loss and the risk of environmental pollution [23,24]. Accordingly, real-time on-site monitoring and post-contamination detoxification are the most effective solutions to mycotoxin contamination. Small peptides have various advantages, such as mature synthesis protocols, diverse structures, and excellent biocompatibility, and share the same chemical structure as proteins, which are widely used in biological, pharmaceutical, chemical, and other fields [25,26]. Meanwhile, with the application of small peptide-based immunological assays in mycotoxin detection and the development of peptide-based artificial enzymes with mycotoxin degradation potential, small peptides have promising applications in mycotoxin control [27,28]. This review mainly summarizes the feasible applications of small peptides in mycotoxin detection, peptide-based artificial enzymes, and their potential applications in mycotoxin removal, to provide new ideas for the detection and control of mycotoxins (Figure 1).

## 2. Design and Screening of Small Peptides

Peptide-based sensors and artificial enzymes have unique advantages and broad application prospects in mycotoxin control. To quickly obtain ideal small peptide sequences, it is crucial to develop convenient small peptide construction methods that can be applied to various scenarios. The main approaches currently used to design and screen small peptides with affinity to mycotoxins are phage display peptide libraries, artificial chemical synthesis, and rational design [29].

### 2.1. Phage Display Peptides

In 1985, Smith used genetic engineering to insert a foreign gene into the genome of filamentous bacteriophage to display the peptide encoded by the target gene as a fusion protein [30]. Subsequently, he cloned the synthesized oligonucleotide fragment with random sequence into filamentous bacteriophage to display one peptide on the surface of each bacteriophage particle after expression [31]. All these phages displaying different peptides constitute a library of phage display peptides, from which binding peptides to specific proteins were obtained by screening. Since it directly related the phage expression peptide to the coding gene, the DNA sequence can be easily obtained after amplification and cloning, thus establishing a random peptide library for phage surface display [32,33]. Therefore, phage display technology can display small peptides on the surface of phage rods, either on the backbone or on both ends, which can be achieved by the targeted insertion of a DNA sequence encoding foreign proteins or peptides [34]. Furthermore, the small peptides displayed on the phage surface can still recognize molecularly targeted binding sites.

**Figure 2:**
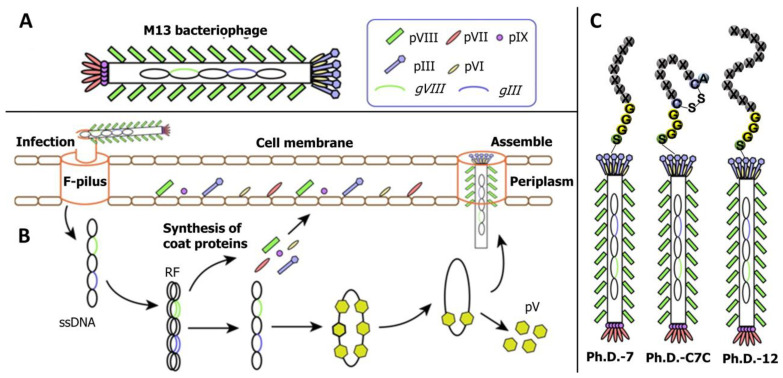
The basic structure and life cycle of the M13 bacteriophage. (**A**) Structure of the M13 bacteriophage; (**B**) the life cycle of the M13 bacteriophage; (**C**) commercial M13 bacteriophage display peptide library. RF: Replicate form; pV: Phage protein.

The phage display technology won the 2018 Nobel Prize in Chemistry, and is widely used for antigen–antibody library building, drug designing, vaccine research, pathogen detection, gene therapy, antigen epitope research, and cell signaling research. Although several phage display techniques have been developed, M13 filamentous phage is the most commonly used in screening small peptides corresponding to mycotoxins. M13 phage is an *Escherichia coli*-specific filamentous phage with a circular single-stranded DNA genome containing 11 genes, 5 of which are used to encode coat proteins (pIII, pVI, pVII, pVIII, and pIX), and its structure is illustrated in Figure 2A [34]. According to the location of the exogenous peptide chain insertion, the M13 phage display system could divide into pIII-mediated display and pVIII-mediated display. The process of filamentous phage adsorption, invasion, proliferation, and release is illustrated in Figure 2B [34]. The M13 phage can produce massive progenies in a short period without killing the host, which varies between the lytic and lysogenic phages. Currently, the main commercial phage display peptide library kits commonly used are loop-constrained heptapeptide library (Ph.D.-C7C), linear heptapeptide library (Ph.D.-7), and linear dodecapeptide library (Ph.D.-12) (Figure 2C). A random peptide library may display tens of millions of peptide epitopes, which can quickly make these phages bind to specific target molecules by bio-panning steps, and then the sequence of phage display peptide can be determined by gene sequence analysis; finally, the sequence of small peptides that has affinity with the target molecule can be obtained [35].

### 2.2. Combinatorial Peptides

Combinatorial chemistry is a set of synthetic strategies and screening protocols that link building blocks, such as amino acids, nucleotides, and organic small molecules, in a combination to synthesize a chemical library containing a large number of compounds and screen them for compounds with some physical, chemical, or pharmacological activity [36,37]. The combinatorial peptide library technology originated from combinatorial chemistry, which has been widely used to screen novel affinity ligands that can be used as materials for affinity chromatography or as new receptors for sensors and biosensors [29,38]. In the combinatorial peptide library, 20 natural amino acids can be used as building blocks, and the size of the library is determined by the number of amino acids (n) that constitute a peptide of a specific length, which can be simply calculated as 20^n^. If a dipeptide library is constructed, there are 400 (20^2^) combinations, a tripeptide library has 8000 (20^3^) combinations, …, and an octapeptide will have 25,600,000,000 (20^8^) combinations.

**Figure 3:**
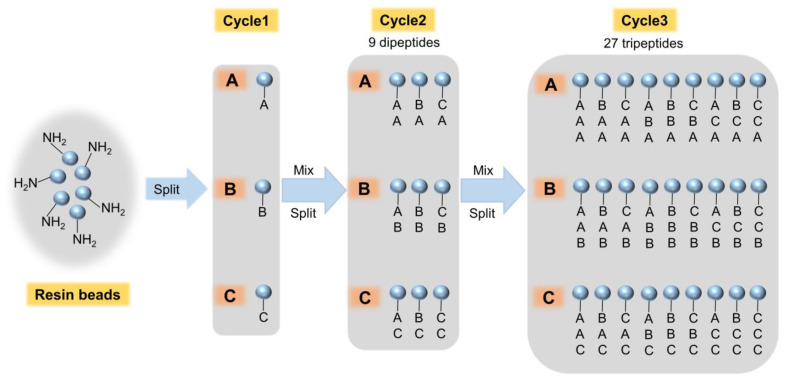
The one-bead one-compound combinatorial peptide library. A, B, and C represent any of the amino acids.

The phage display peptide library method is simple and economical, but this biological method is limited to peptides containing only L-amino acids. However, synthetic combinatorial libraries can accommodate D-amino acids, non-natural amino acids, and even organic molecules, which makes these methods highly general synthetic peptide libraries. The most commonly used method for constructing combinatorial peptide libraries is the one-bead one-compound library (OBOC) [38,39] (Figure 3). Lam et al. [40] first reported a pentapeptide library synthesized by resin beads (100–200 μm) as a carrier, in which only one pentapeptide is coupled to each resin bead. These pentapeptides can bind to the corresponding proteins, using specific proteins such as antibodies, ligands, and enzymes labeled with fluorescein or isotopes as probes. The specific pentapeptide can finally be screened and isolated from the peptide library based on these markers [40]. Giraudi et al. constructed a hexapeptide library by OBOC combinatorial synthesis and screened out a hexapeptide (SNLHPK), showing good affinity (K_eq_ = 3.4 × 10^4^ M^−1^) towards ochratoxin A (OTA). This hexapeptide can be used for the solid-phase extraction of OTA from wine and quantification of OTA in wine samples at concentration levels down to 0.10 μg/L [41]. The application of combinatorial chemistry in small-molecule drug screening is widespread [37,42]. Recently, research on DEL technology that combines DNA coding and combinatorial chemistry has shown explosive growth and has revolutionized small-molecule drug development, which may have broader use in mycotoxin detection [43].

### 2.3. Rational Design Peptides

**Figure 4:**
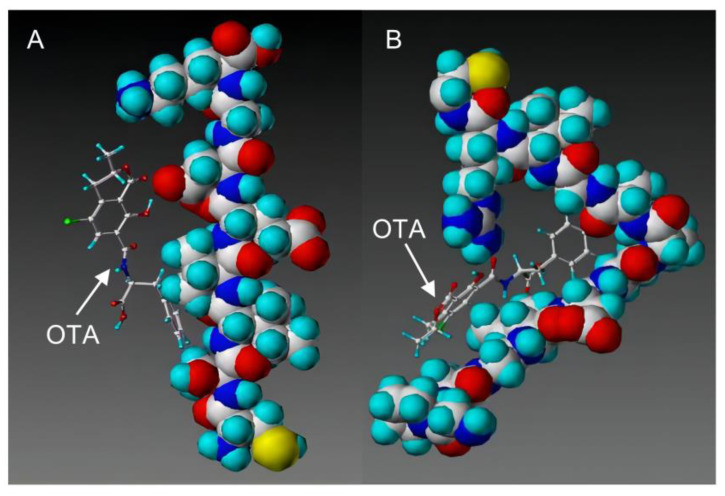
Rationally designed peptide receptor binding with OTA ((**A**): GPAGIDGPAGIRC; (**B**): CSIVEDGL) [13]. Peptide sequences are represented as space-filled, and OTA and AFB_1_ as stick and ball structures.

With the development of high-performance computing (HPC) and further knowledge of the structure and function of peptides, rational design strategies have achieved remarkable progress, and at present, we can accurately evaluate the three-dimensional structure of proteins based on the primary peptide sequences [44,45]. Therefore, there are increasingly small peptides synthesized based on rational design and generally divided into two categories. The first one is based on molecular modeling and docking techniques, and the small peptides are designed and screened by rational analysis. By evaluating the affinity of common amino acids with target molecules, the amino acids with higher affinity among them were selected to design potential small peptides with the help of software, and finally the best small peptide sequence was determined by artificial synthesis and experimental verification. Using this strategy, Parker and Heurich designed and screened small peptide sequences with specific affinity to AFB_1_ and OTA, respectively, and demonstrated through synthesis and analysis that these small peptides can be used in the rapid detection of AFB_1_ [46] and OTA [13,47] (Figure 4).

As increasingly functional small peptides are mined and designed, there are various databases of small peptide sequences with different functions that can be easily retrieved. Therefore, another frequently rational design method for small peptides is to retrofit the already discovered small peptides. Although the affinity of the peptide synthesized by rational design is slightly imperfect, the peptide sequence can be optimized to improve its properties based on conformational relationships [48]. Currently, rationally designed small peptides are widely applied in the monitoring of disease biomarkers [49], pathogenic microbes [50], public hazardous [51], etc. Meanwhile, rationally designed peptides coupled with gold nanomaterials have gained` increasing interest in biosensors, cell imaging, drug delivery, and therapy, which have promising applications in medical and on-site detection [52].

## 3. Small Peptides in Mycotoxin Detection

Peptides have emerged as a promising approach to synthetic biomimetics [53]. The excellent properties of synthetic peptides in small-molecule contaminant (SMCs) recognition make them a potential alternative to antibodies and natural receptors in mycotoxin biosensor applications [54]. Small peptides show high-affinity binding to these small analytes and have stability, playing an increasingly important role in the rapid detection of mycotoxins [55]. Among immunologically based mycotoxin detection methods, peptides can serve as competing antigens, coating antigens, and anti-immune complexes (Table 2).

### 3.1. Peptides as Competing Antigens

**Figure 5:**
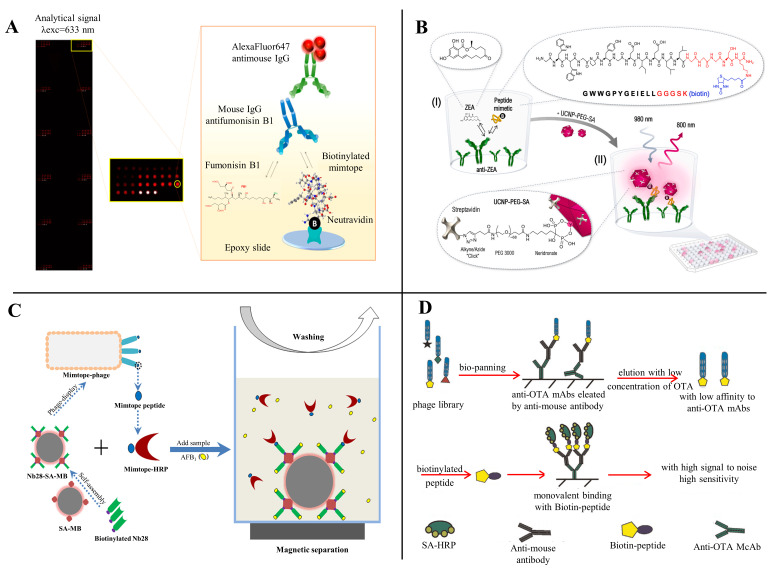
Peptides as competing antigens for mycotoxins detection. (**A**) FB_1_ detection; Reprinted with permission from Ref. [77]. 2017, Anal. Chem. (**B**) ZEN detection; Reprinted with permission from Ref. [73]. 2020, Biosens. Bioelectron. (**C**) AFB_1_ detection; Reprinted with permission from Ref. [62]. 2019, Talanta. (**D**) OTA detection; Reprinted with permission from Ref. [60]. 2016, Talanta.

As stated earlier, competitive antigens are essential in competitive immunoassays, but the obtaining of specificity antigens always involves the use of mycotoxins. Peptides can serve as competing antigens and competitively bind with specific monoclonal antibody (mAb), which can avoid the use of poisonous mycotoxins [62,65]. In 1999, Yuan et al. [83] identified two phage-displayed mimotopes (SWGPFPF and SWGPLPF) and used them to detect DON firstly. Subsequently, mimotopes as competitive antigens for the detection of mycotoxins have been increasingly reported, involving OTA, AFB_1_, ZEN, FB_1_, and other mycotoxins [84]. Peltomaa et al. [77] reported the selection of a novel dodecapeptide (VTPNDDTFDPFR) from a 12-mer peptide library; then, a biotinylated synthetic derivative of this mimotope (VTPNDDTFDPFRGGGSK-Biotin) was used for the detection of FB_1_ by a competitive binding inhibition assay. Its 50% inhibitory concentration (IC_50_) was 37.1 ng/mL, with a detection limit (LOD) of 11.1 ng/mL, and a dynamic range from 17.3 to 79.6 ng/mL (Figure 5A). Recently, the authors used the same method to obtain a ZEN mimetic epitope peptide (GWWGPYGEIELL); the peptide was used to create fusion proteins with the bioluminescent Gaussia luciferase (GLuc) that were directly used as tracers for mycotoxin detection in a competitive immunoassay [72]. Meanwhile, using the same small peptide sequence developed a competitive upconversion-linked immunosorbent assay (ULISA) for ZEN monitoring with a LOD of 20 pg mL^−1^ [73] (Figure 5B).

Chen et al. [69] constructed a peptide@Tyr-RMC probe by selecting a mimotope peptide from a phage display library and labeling it a Tyr-RMC composite, which was used to develop a competitive ELISA for the ultrasensitive detection of ZEN. It has a linear range of 10^−6^–1.0 ng/mL, and a low detection limit of 10^−6^ ng/mL. Zhao et al. [62], using AFB_1_ as a model system, selected a mimotope (YSWHEWYIPQLS) from Ph.D-12 phage display peptide library, and the rapid magnetic-beads-based directed competitive ELISA (MB-dcELISA) was developed by mimotope ME17. The IC_50_ and LOD of the MB-dcELISA were 0.75 and 0.13 ng/mL, respectively, with a linear range of 0.24–2.21 ng/mL (Figure 5C). Zou et al. [60] obtained a mimotope from the commercial Ph.D.-7 phage display peptide library and connected it with biotin (GMVQTIF-GGGSK-biotin). The biotinylated 12-mer peptide was used as a competing antigen to develop a competitive peptide ELISA for OTA detection and showed a wide linear range of 0.005–0.2 ng/mL with the detection limit of 0.001 ng/mL. The IC_50_ was 0.024 ng/mL, which is approximately five times more sensitive as a competing antigen than the OTA-HRP conjugates used in the conventional ELISA (Figure 5D).

### 3.2. Peptides as Coating Antigens

**Figure 6:**
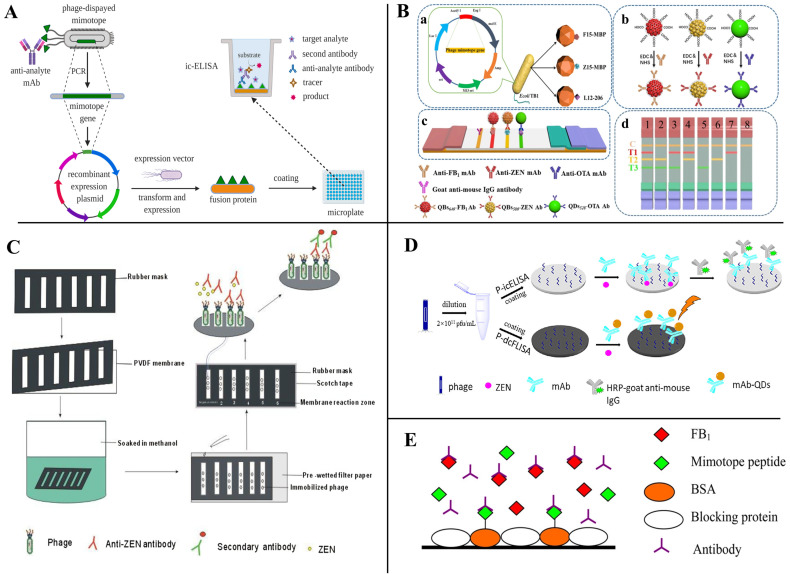
(**A**) Schematic of mimotope fusion protein as coating antigens for mycotoxin detection; Reprinted with permission from Ref. [11]. 2021, Food Chem. (**B**,**C**) Mimotope fusion protein as coating antigens for mycotoxin detection; (**a**) Biological expression strategy of peptide-MBP fusion protein, (**b**) Fabrication process of the prepared QDs/QBs-mAb probes, (**c**) schematic illustration of the tricolor mICA, (**d**) schematic illustration for the interpretation of test results; Reprinted with permission from Ref. [67], 2014, Food Control, and Ref. [86], 2020, J. Agric. Food. Chem. (**D**,**E**) Peptides as coating antigens for mycotoxin detection; Reprinted with permission from [74], 2022, J. Food Saf., and Ref. [75], 2013, J. Agric. Food. Chem.

Peptides also can serve as competing antigens in immunological-based mycotoxin detection. He et al. [67] selected a phage display dodecapeptide (ESYWATVPWTRH) as a substitute for coating antigens and applied it for the rapid detection of ZEN by dot-immunoassay (Figure 6C). The cut-off level for detecting ZEN in cereal samples was 50 mg/kg and the results can be accomplished within 10 min. With the phage display peptide library technology, Zhou et al. [74] reported a phage mimotope-based direct competitive fluorescence immunosorbent assay (P-dcFLISA); the IC_50_ of P-dcFLISA was 0.301 ng/mL, which was lower than the phage-based indirect competitive enzyme-linked immunosorbent assay (P-icELISA) under the same conditions (Figure 6D). The LOD and detection range of P-dcFLISA was 0.023 ng/mL and 0.060–1.531 ng/mL, respectively. However, there is no corresponding amino acid sequence information in the article. Liu et al. [75], utilizing mimotope peptide-bovine serum albumin conjugate as a coating antigen, developed a peptide ELISA for detecting FB_1_, in which the IC_50_ and LOD were 6.06 ng/mL and 1.18 ng/mL, respectively (Figure 6E). Except for phage display peptides, small synthetic peptides by rational design are also used as coating antigens for mycotoxin detection. Bazin et al. [85] designed an OTA-binding peptide (VYMNRKYYKCCK) derived from an oxidoreductase and developed a peptide-based competitive enzyme-linked immunosorbent assay (peptide-based competitive ELISA) in which the peptide was the coating antigen.

Mimotope-based fusion proteins can also be used as synthetic coating antigens for the detection of mycotoxins [11] **(**Figure 6A). Xu et al. [76], using FB1 as a model hapten, screened two mimotopes (F1: NNAAMYSEMATD, F15: TTLQMRSEMADD) that have affinity to the anti-FB1 antibody from a 12-mer peptide library and developed a new method for the development of a sensitive and environmentally friendly immunoassay for FB1 based on the peptide–MBP (maltose-binding protein) fusion protein. Quantitative immunoassay for FB1 using F1-MBP and F15-MBP showed the LOD was 0.32 and 0.21 ng/mL, respectively, and the IC_50_ of the assay was 2.15 and 1.26 ng/mL, which was 10 times more sensitive than the conventional FB1-BSA conjugate-based ELISA. Meanwhile, using the same strategy, they also constructed a small peptide–MBP fusion protein-coated antigen that can be used to detect OTA and DON with good sensitivity and stability [58,66]. Recently, to monitor the co-contamination of mycotoxins in agricultural products and foods, Yan et al. [86] developed a mimotope–MBP fusion protein-based multiplex immunochromatographic assay (mICA) that can quickly and simultaneously detect FB1, ZEN, and OTA without the building-up process of mycotoxin conjugates. The LOD of peptide–MBP-based mICA for FB1, ZEN, and OTA were 0.25, 3.0, and 0.5 ng/mL, respectively (Figure 6B).

### 3.3. Anti-Immune Complex Peptides

As small molecule contaminants (SMCs), mycotoxins usually only have one immunological binding site and are not suitable for detection by conventional sandwich non-competitive immunoassays [87]. Alternatively, the development of non-competitive immunoassays using specific recognizers for the immune complex of anti-SMC antibodies is a feasible strategy. Anti-immune complex peptide (AIcP) is a peptide that specifically binds to immune complexes, neither antibodies nor antigen monomers [88]. Biopanning the phage display random peptides library using antigen–antibody conjugates allows the screening of peptides that can bind specifically to immune complexes and can be used to establish non-competitive immunoassays [89]. Unlike mimotopes, anti-immune complex peptides recognize only complexes of antigens and antibodies and do not bind to antibodies or antigens alone, and because of this dual site recognition pattern, anti-immune complex peptides are often thought to improve the specificity of assays.

**Figure 7:**
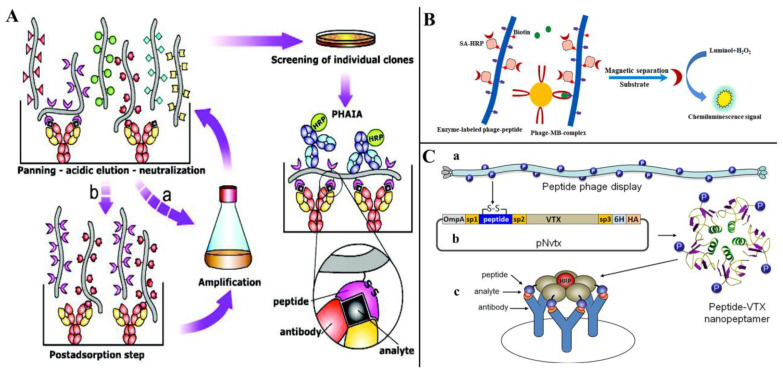
Peptides as anti-immune complexes for small-molecule contaminant detection (SMC). (**A**) The process of panning anti-immune complex phages and establishing phage anti-immune complex assay (PHAIA). Reprinted with permission from Ref. [90]. 2007, Anal. Chem. (**B**) Noncompetitive magnetic-phage anti-immune complex immunoassay (Nc-MCLEIA) for AFB_1_ detection. Reprinted with permission from Ref. [61]. 2022, Food Chem. (**C**) Multivalent display anti-immunocomplex peptides on verotoxin for clomazone detection [92]; (**a**). The anti-immuncomplex peptide selected from phage libraries, (**b**). Peptide coding sequence cloned into the pNvtx vector and fused to the VTX gene, (**c**). Recombinant nanopeptamer conjugated to peroxidase to detect the formation of the immunocomplex.

González-Sapienza’s team achieved promising results in the detection of pesticide contaminants using anti-immune complex peptides, demonstrating the advantages of the method in the detection of SMCs [82,89,90,91], and the process of panning anti-immune complex phages and establishing the phage anti-immune complex assay (PHAIA) is depicted in Figure 7A [90]. Zou et al. [61], using AFB_1_ and anti-AFB_1_ nanobody conjugates as the immune complex, screened anti-immune complex peptides from a phage display random linear 8-mer peptide library; the best binding peptide was biotinylated and coupled with horseradish peroxidase-labeled streptavidin (SA-HRP) for developing the magnetic-phage anti-immune complex immunoassay (MPHAIA) detection of AFB_1_ (Figure 7B). Phage-peptide p13 (DLLWVPST)-based MPHAIA, showing the lowest SC_50_ (50% signal saturation concentration) value (0.12 ng/mL) and the highest OD_max_/SC_50_ ratio (12.75), was selected for further study. Under the ultimate condition, the LOD was 0.006 ng/mL, with a linear range of 0.019–0.407 ng/mL. Lassabe et al. [92] used a verotoxin (VTX) of *Escherichia coli* as a scaffold for multivalent display anti-immunocomplex peptides, and, among these peptides, ICX09m (CLEAPNVEAC) showed 10-fold increased sensitivity and excellent recovery than the competitive ELISA for clomazone detection (Figure 7C).

Other studies use this method for residual pesticide and veterinary drug detection, but with few applications in mycotoxin detection. Although the non-competitive assay has advantages, the technique is still in the early stages of development, and the biggest challenge in developing this assay is the screening of anti-immune complex peptides. Therefore, improving the success rate of peptide screening for anti-immune complexes will significantly enhance the development of non-competitive immunoassays for small-molecule compounds, such as mycotoxins and pesticides. The anti-immune complex peptides of mycotoxins, pesticides, and other small molecules are summarized in Table 2.

## 4. Small Peptides in the Construction of Artificial Enzymes

**Figure 8:**
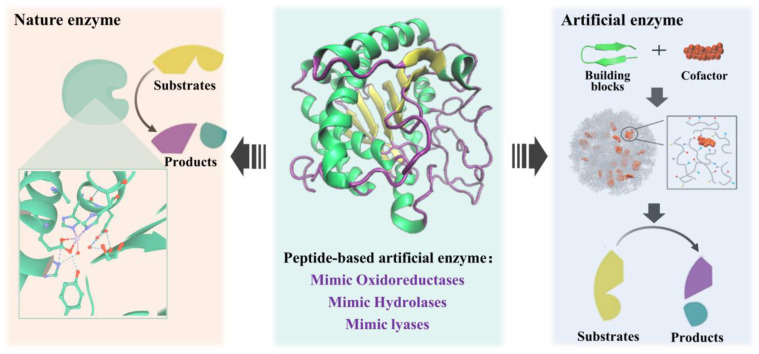
Artificial enzyme construction process by mimicking nature enzymes.

Small peptides are the ideal material to construct artificial enzymes because of the advantages of high similarity to natural enzymes and controllable structure [93,94]. The high efficiency of the catalytic activity of natural enzymes comes from two main aspects: (1) the active cavity that can just accommodate the substrate molecule; and (2) the attack of the catalytically active amino acid on the substrate and the stabilization of the transition state. Peptide-based artificial enzyme construction is mainly inspired by the structure and catalytic mechanism of the natural enzyme active site, and supramolecular catalysts with enzyme-like active sites can be generated by self-assembly of small-peptide molecules or supplemented with other supporting structures to mimic the natural enzyme active center (Figure 8).

Peptide-based artificial enzymes have been proven efficient in the degradation of plasticizers [95], phenolic contaminants [96,97], Azo dyes [98], cellulose [99], and the treatment of Alzheimer’s disease [100]. Despite the few relevant reports on mycotoxin removal by peptide-based artificial enzymes at present, it may provide a potential new approach for mycotoxin control in the future. The peptide-based artificial enzymes that have been reported mainly mimic the catalytic activities of oxidoreductases, hydrolases, and lyases (Table 3).

### 4.1. Peptide-Based Oxidoreductase Mimics

**Figure 9:**
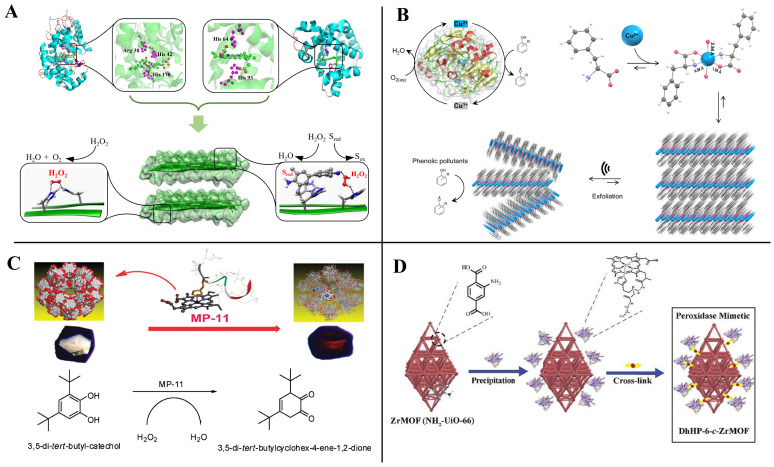
Peptide-based oxidoreductase mimics with natural heme-containing enzyme activity. (**A**) Laccase activity. Adapted from Ref. [101]. (**B**) Peroxidase activity [97]. (**C**,**D**) Immobilized into a mesoporous metal-organic framework. Reprinted with permission from Ref. [96], 2020, Catal. Commun., and Ref. [136], 2011, J. Am. Chem. Soc.

Small peptides can mimic oxidoreductase activities, such as laccase [97], peroxidase [101], and superoxide dismutase [132], alone or in combination with cofactors. Begum et al. [133], first using copper (II)-peptides mimicking the active site of laccase from *Trametes versicolor* (PDB: 1KYA), designed artificial enzymes that can catalyze oxidation and polymerization to degrade aromatic and cyclic pharmaceutically active compounds (PhACs). Tp-CuS 12 was demonstrated to be the best catalyst in degrading the cresols, 2,4-dichlorophenol, and ibuprofen, and revealed potential applications in environmental remediation. More recently, similarly inspired by the laccase catalytic site, Makam et al. [97] designed a simple, efficient, and robust phenylalanine-based single amino acid artificial enzyme (Figure 9B). It can catalyze the rapid oxidation of phenolic pollutants, which were approximately 5400-fold more cost-effective, 36-fold more sensitive, and 4-fold more efficient than a natural laccase enzyme. In addition to the rational design of copper-binding peptides with oxidoreductase activity, Xin et al. [134] found that the natural copper-binding peptide (Methanobactin, Mb) from *Methylosinus trichosporium* OB3b exhibited peroxidase activity; further studies showed that the catalytic efficiency of Mb-Cu was significantly enhanced by immobilizing it on gold nanoparticles (AuNPs).

Microperoxidase-11 (MP-11) is a heme-containing peptide from Cytochrome c by proteolytic digestion [135]. The molecular weight of MP-11 is much smaller than that of natural peroxidase, so it has better solubility in organic solvents. Accordingly, Wariishi et al. [98] found that MP-11 exhibited effective decolorization activity against both azo and anthraquinone dyes in 90% methanol. Subsequently, Lykourinou et al. [136] and Ding et al. [96] immobilized MP-11 and MP-11 mimic (DhHP-6) in a metal-organic framework to improve their catalytic rates, respectively, and found that the catalytic properties of the newly constructed artificial enzymes were both improved, among which DhHP-6-c-ZrMOF could be a promising catalyst for the efficient degradation of phenol pollutants (Figure 9C,D).

Natural redox enzymes (e.g., peroxidase, laccase, and glucose oxidase) mainly rely on the precise arrangement of amino acid residues near the active site and cofactor to catalyze the redox reaction of the substrate efficiently, among which the cofactor plays a key role in electron transfer. However, when the temperature increases or pH fluctuates, the spatial arrangement of amino acid residues in the active site changes, leading to the movement, shedding, or aggregation of cofactors and resulting in the irreversible denaturation and inactivation of the enzyme. Based on the catalytic properties of histidine side chain imidazole, Liu et al. [101] designed polyhistidine peptides of different lengths (number of amino acids from 2 to 20), which were assembled to form large two-dimensional nanosheets or ribbons through hydrogen bonding, charge, or *π–π* stacking interactions, and the sizes reached up to millimeters. The catalytic activity H15 exhibited significant activity in catalyzing the oxidation of 3,3′,5,5′-tetramethylbenzidine (TMB), homovanillic acid (HVA), and nicotinamide adenine dinucleotide (NADH) by H_2_O_2_ in the absence of a heme cofactor and metal conditions (Figure 9A).

### 4.2. Peptide-Based Hydrolase Mimics

According to the degradation of the different chemical bonds, peptide-based artificial hydrolases mainly be used to catalyze ester bonds, peptide bonds, and glycosidic bond hydrolysis. Among them, ester bond degrading artificial enzymes have been reported more frequently. Esterases are enzymes that catalyze the rapid cleavage of ester bonds and the hydrolysis of esters to the corresponding alcohols and acids. It is commonly found in living organisms and widely used in food, medicine, chemistry, biology, and other fields. Meanwhile, the serine protease catalytic center amino acids histidine (His), aspartate (Asp), and serine (Ser), also known as the catalytic triad, are often introduced into peptide sequences to construct esterase mimics [137]. In addition, numerous studies have reported that histidine can act as an artificial enzyme active site to catalyze the hydrolysis of esters [101,138]. The main ester substrates that have been reported to be able to be catalyzed by peptide-based ester-bond-degradation artificial enzymes are *p*-nitrophenol acetate (*p*NPA) [121], *p*-nitrophenol propionate (*p*NPP) [112,121], *p*-nitrophenol butyrate (*p*NPB) [113], *p*-nitrophenyl acetate methyl ester (*p*-NPMA) [112], dioctyl phthalate (DEHP) [139], uridine 3’-(2,2,2-Trichloroethylphosphate) [140], acetyltyrosine ethyl ester (ATEE) [141], benzoyltyrosine ethyl ester (BTEE) [141], Cbz-Phe-ONP [142], BAPNA [122], etc.

**Figure 10:**
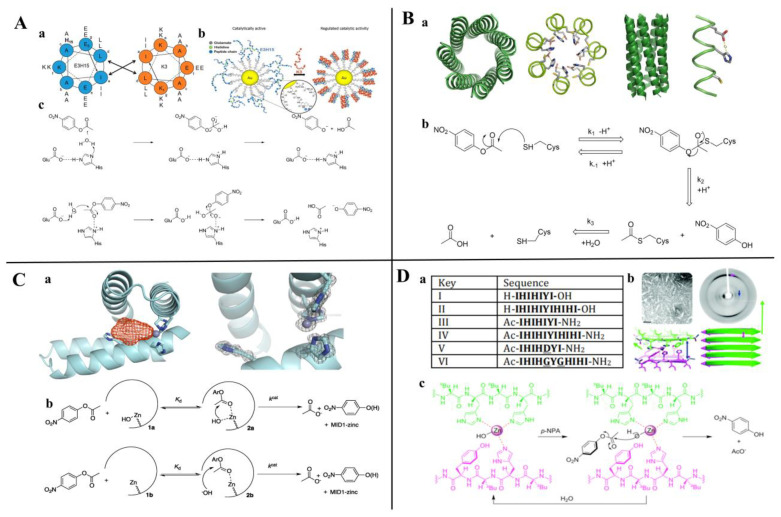
Peptide-based hydrolase mimics with esterase activity. (**A**) immobilized onto gold nanoparticles; (**a**) Peptide sequences of E3H15 and K3, (**b**) The structure of Au@E3H15 and mechanism for regulated catalytic activity, (**c**) Proposed mechanism for the hydrolysis of *p*NPA catalyzed by E3H15 and Au@E3H15 monolayer. Reprinted with permission from Ref. [108]. 2017, Biomacromolecules. (**B**) having catalytically active Cys-His-Glu triads by a de novo design; (**a**) The structure of CC-Hept-Ile-Cys-Ile, (**b**) Proposed mechanism for the reaction of CC-Hept-Cys-His-Glu with *p*NPA via a thioester intermediate. Adapted from Ref. [143]. (**C**) having a small cleft and open zinc coordination site; (**a**) The structure of MID1-zinc, (**b**) Proposed mechanism for the reaction of MID1-zinc with *p*NPA. Reprinted with permission from Ref. [121]. 2012, Biochemistry. (**D**) capable of self-assembling into amyloid structures; (**a**) Peptide designs, (**b**) The structure of peptide III, (**c**) The proposed mechanism of hydrolysis for the substrate *p*NPA by fibrils of peptide III. Reprinted with permission from Ref. [118]. 2017, Nanoscale.

Mikolajczak et al. [108] reported a peptide-gold nanoparticle coupling Au@E3H15 (E3H15, H_2_N-CGGYE_5_IAALEKEIAH_15_LEKEIAALEK-CO_2_H) with esterase properties by rational design, in which the catalytic activity is regulated by peptide conformational changes and can be significantly inhibited by the addition of small peptides with complementary α-helical structures (Figure 10A). Burton et al. [143] completed a de novo design of a peptide-based artificial hydrolase using the thermostable α-helical barrel, which can catalyze the hydrolysis of *p*-nitrophenyl acetate. Eventually, CC-Hept-Cys-His-Glu was heptameric in a solution, confirmed by the X-ray crystal structure (PDB: 5EZC) and showing the best activity with *K*_M_ and *k*_cat_/*K*_M_ at 134 μM and 3.7 M^−1^ s^−1^, respectively (Figure 10B). Similarly, Der et al. [121] de novo designed an artificial enzyme MID1-zinc (PDB: 3V1C) with the ability to catalyze *p*NPA and *p*NPP hydrolysis (Figure 10C).

Garawi et al. [118] designed a series of small peptide sequences capable of self-assembling into amyloid fibrils through the rational design approach (Figure 10D). Catalytic activity studies showed that III, IV, and VI were able to catalyze the degradation of *p*NPA, sequence III (Ac-IHIHIYI-NH_2_), showing the best catalytic activity with a *k*_cat_/*K*_m_ of 355 M^−1^s^−1^. The catalytic mechanism may be that the water molecule activates the Zn^2+^–imidazole group complex, forming a tetrahedral structure that acts directly with the carboxyl group of *p*NPA to form an acyl-Zn-imidazole complex; the tyrosine in the sixth position assists the protonation process of the zinc ion–imidazole group complex. The successful construction of these artificial hydrolases provides evidence for understanding the evolution of natural enzymes and an effective strategy for the rational design of enzymes in the future.

In addition to mimicking esterase activity, peptide-based artificial enzymes can also be used to catalyze the degradation of peptide bonds and glycosidic bonds. Gao et al. [122] used the synergistic interaction of POMD (polyoxometalate with Wells-Dawson structure) and 7 peptides (N-His-Sar-His-Sar-His-Sar-His, Sar = sarcosine) to mimic natural trypsin activity. AuNPs were used to facilitate electron transfer between heptapeptides and POMD, and could also serve as a backbone for the artificial enzyme. POMD can be linked to the surface of AuNPs; the 7 peptides can also be immobilized on the surface of AuNPs by adding cysteine at the N-terminal, which finally constitutes a multifunctional artificial enzyme (AuNPs@POMD-8pep). The artificial enzyme has both protease activity and superoxide dismutase activity, and the specific activity was 8.80 × 10^5^ U/mg using BAPNA as substrate, which was much higher than that of natural trypsin (5.14 × 10 U/mg) under the same conditions. He et al. [144] designed a series of β-folded heptapeptides containing multiple glutamic or aspartic acids, based on the self-assembly properties of small molecule peptides. All these small peptides were repetitive patterns of polar and nonpolar amino acids, consistent with the structural periodicity required for amyloid structure formation. After self-assembly, most of them were found to have measurable hydrolytic activity of cellobiose, with PC5 (Ac-FEFEAEA-CONH_2_) having the best catalytic activity. The factors affecting the catalytic activity are the amount of glutamate, the conformation of the small peptide backbone, the distribution of intramolecular hydrogen bonds, the spatial resistance to substrate, and the crystal state of the peptide nanofibers.

### 4.3. Peptide-Based Lyase Mimics

**Figure 11:**
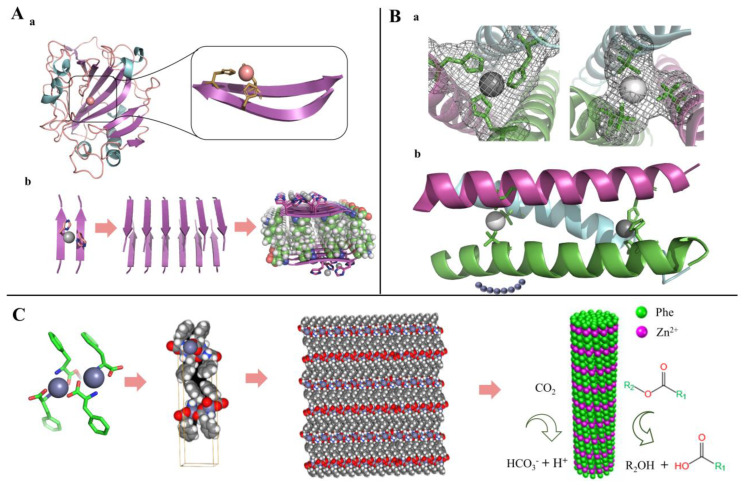
Peptide-based lyase mimics inspired by natural human carbonic anhydrase. (**A**) Peptide-based artificial enzyme capable of self-assembling into nanofibers; (**a**) Structure of human carbonic anhydrase showing a typical metal-binding motif, (**b**) Structure and assembly process of artificial enzyme. Adapted from Ref. [131]. (**B**) Artificial protein containing two separate metal sites by a de novo design; (**a**) Top-down view of the structural trigonal thiolate site (right) and side view of the tetrahedral catalytic site (left), (**b**) One of two trimers found in the asymmetric unit of the crystal structure. Adapted from Ref. [107]. (**C**) single phenylalanine self-assembling into needle-like architectures with carbonic anhydrase activity. Reprinted with permission from Ref. [93]. 2020, ACS Catal.

Lyases are enzymes that cleave C–C, C–O, C–N and other bonds by elimination to form multiple bonds or rings. Small peptides can mimic the reaction of the corresponding substrate catalyzed by aldolase and carbonic anhydrase [116,130]. Rufo et al. [131] designed a series of heptapeptides capable of self-assembling into amyloid structures and investigated whether they have enzyme-like catalytic effects. The results showed that, with the assistance of zinc ions, these self-assembled short peptides possess esterase activity and can catalyze the hydrolysis of *p*NPA. Among them, Ac-IHIHIQI-CONH_2_ showed the best catalytic activity; the *K*_M_, *k*_cat_, and *k*_cat_/*K*_M_ were 0.4 mM, 2.6 × 10^−2^ s^−1^, and 62 M^−1^ s^−1^, respectively (Figure 11A). With the TRI family peptide (Ac-G(LKALEEK)_4_G-NH_2_) as a scaffold, Zastrow et al. [107] developed a true de novo metalloenzymes based on a designed catalytic metal site and a defined three-dimensional structure. [Hg(II)]_S_[Zn(II)(H_2_O/OH^-^)]_N_(TRIL9CL23H)_3_^n+^ catalyzes *p*NPA hydrolysis only ∼100-fold less than human carbonic anhydrase (CA)II, and the CO_2_ hydration occurs with efficiency within approximately 500-fold of CAII (Figure 11B).

Phenylalanine has both positively as well as negatively charged groups and aromatic groups, and these properties allow phenylalanine to self-assemble to form specific nanostructures through hydrogen bonding, *π–π* stacking, and hydrophobic interactions. Abramovich et al. [145] found that phenylalanine can spontaneously self-assemble into supramolecular crossed β-sheet secondary structures with amyloid characteristics. Later, Makam et al. [116] constructed an artificial enzyme for the first time by self-assembling single phenylalanine with zinc ions to form a nanoneedle structure. The crystal structure diffraction analysis showed that one zinc ion was bound to two phenylalanines via amine and carboxylate groups; the layers were stabilized by *π–π* interactions. Activity validation showed that the artificial enzyme has both esterase and carbonic anhydrase activities, which is the smallest artificial hydrolase with the lowest molecular weight found to date, and the catalytic activity (*k*_cat_/*K*_M_ = 46 × 10^−2^ (gl^−1)−1^ s^−1^) was 8-fold higher than that of natural carbonic anhydrase (*k*_cat_/*K*_M_ = 5.7 × 10^−2^ (gl^−1)−1^ s^−1^) based on mass (Figure 11C).

Aldolase is a lyase that catalyzes the reversible conversion of fructose-1,6-bisphosphate to glyceraldehyde, 3-phosphate, and dihydroxyacetone phosphate. Tanaka and Carlos [129] tried to select aldolase peptides using an 18-residues peptide (YLK-18-opt) that can catalyze oxaloacetate decarboxylation. To study the catalytic activity of this peptide, a six-peptide library was designed at its C-terminus and displayed on the phage. The small peptides were obtained by panning phage display peptide library (YKLLKELLAKLKWLLRKLXXXXXX, X = natural 20 amino acids) that bound to substrate. Among the peptides, FT-YLK3 (YKLLKELLAKLKWLLRKLLGPTCLNH_2_) can catalyze the retro-aldol reaction of the substrate with a *k*_cat_/*k*_uncat_ of 1900 and a *K*_m_ of 1.8 mM. Subsequently, to increase substrate specificity, the authors added the small peptide FluS303 (YPNEFDWWDYYY) at the C-terminal of FT-YLK3 that has affinity to the substrate. The designed FluS303-FTYLK3 exhibits enhanced catalytic properties than FT-YLK3 [130]. More recently, Peme et al. [146] designed a peptide-based artificial enzyme that mimics the catalytic active site of the nature fructose-1,6-bisphosphate aldolase. The peptides exhibited asymmetric aldol reaction catalytic activity for aliphatic and aromatic ketone/aldehydes, and the aldol product yields can reach 44% with excellent enantioselectivity (93%).

### 4.4. Small Peptides in Mycotoxin Removal

**Figure 12:**
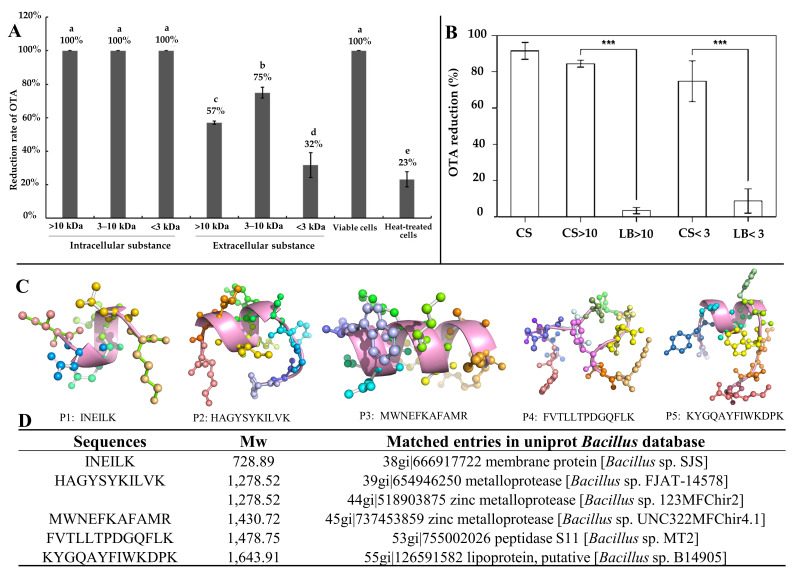
Removal of OTA by microbial-source small molecular substances; (**A**) *Brevundimonas naejangsanensis* ML17 source < 3 kDa ultrafiltration fraction for OTA degradation; Data with different lowercase letters were considered to be significantly different (*p* < 0.05) by Duncan’s multiple comparison test; Reprinted with permission from Ref. [22]. 2022, Food Control. (**B**) *Bacillus subtilis* CW14 source < 3 kDa ultrafiltration fraction for OTA degradation; Bars with *** were significantly different based on ANOVA test (*p* < 0.001); Reprinted with permission from Ref. [21]. 2018, World Mycotoxin J. (**C**) Structure of the small peptide in Figure 12D predicted with PEP-FOLD 3.5 and displayed by pyMOL; (**D**) list of peptides in the *Bacillus subtilis* CW14 < 3 kDa ultrafiltration fraction, identified by LC-ESI-MS/MS using Mascot serve. Reprinted with permission from Ref. [21]. 2018, World Mycotoxin J.

Our team previously isolated a *Bacillus subtilis* CW14 from fresh elk droppings with OTA-degrading ability, and the cell-free supernatant can also degrade OTA efficiently. Interestingly, when the strain culture supernatant was separated by ultrafiltration, both the fractions collected at > 10 kDa (84.9%) and < 3 kDa (74.8%) demonstrated a remarkable ability to remove OTA (Figure 12B) [21]. Then, a carboxypeptidase gene was likely responsible for the OTA degradation by the > 10 kDa fraction was cloned from the *B. subtilis* CW14 genome and has OTA-degrading ability, which was confirmed by expression of the gene in *Escherichia coli*. As for the <3 kDa fraction (CS < 3), the author found the further purified small peptides (0.7 kDa < Mw < 1.7 kDa), showing OTA-degrading activity (45.0%). We speculated that the OTA may be adsorption and removed by some small peptides. Finally, by comparison with the *B. subtilis* protein database, we found five small peptides with molecular weights ranging from 0.73 kDa to 1.64 kDa, which were fragments derived from membrane protein, metalloprotease, zinc metalloprotease, peptidase, and lipoprotein, respectively, and may be present in the CS < 3 fractions as shown in Figure 12C [21]. More recently, we obtained another *Brevundimonas naejangsanensis* ML17 strain from soil, which degraded OTA into OTα and OTB into OTβ with a degrading rate of 100% within 24 h. Similarly, when the ML17 strain intracellular fractions and extracellular components were separated by ultrafiltration. The <3 kDa fractions both degraded OTA within 24 h at a rate of 100% and 31.64% [22] (Figure 12A).

Small peptides derived from microorganisms have diversity functions [147], based on the following evidence: (1) small peptides derived from *Methylosinus trichosporium* (Methanobactin, Mb) possess peroxidase activity [134]; (2) small peptide fragments derived from natural Tob1 protein (JAL-TA9 and ANA-TA9) can catalyze peptide bond degradation [124,126,127]; (3) heme-containing small peptides derived from horse heart cytochrome c (Microperoxidase-11, MP-11) possess peroxidase activity and can catalyze the degradation of azo dyes [98]; and (4) artificial enzymes constructed based on small peptides can mimic a variety of natural enzyme activities and catalyze the degradation of plasticizers [95], phenolic contaminants [96,97], cellulose [99], etc. Thus, we speculated that the small peptides derived from the ML17 strain may have OTA degradation activity. We are currently performing further purification and characterization of these small molecules. Since small peptides have various advantages over natural large molecule proteins, such as simple structure, being easily controlled, having thermal stability, and biocompatibility, this discovery may provide new perspectives for the green and safe removal of mycotoxins in the future.

## 5. Conclusions and Outlook

Small peptides can be constructed and screened in various ways, such as phage display peptide libraries, combinatorial chemistry, and rational design. With the advantages of mature synthesis protocols, diverse structures, excellent biocompatibility, and sharing the same chemical structure as proteins, small peptides are widely used for mycotoxin detection and artificial enzyme construction with mycotoxin degradation potentiality. With further research on structure–activity relationship, synthesis, and screening methods, small peptides are playing an increasingly important role in mycotoxin control.

In mycotoxin detection, small peptides can serve as mycotoxin extraction and clean-up materials to improve the sensitivity and reproducibility of traditional instrument detection. Since traditional methods conduct the real-time detection of mycotoxins in food and foodstuffs with difficulty, immunoassays based on antigen–antibody reactions can achieve rapid and highly sensitive detection. However, these approaches also have problems, such as the need to use mycotoxin standards and the difficulty and complexity of anti-toxin antibody preparation, while small peptides can be used as coating antigens, competitive antigens, anti-immune complex peptides avoiding the use of toxin standards, or simplifying antibody preparation, which have an important role in the detection of mycotoxins. Meanwhile, the application of peptide-based biosensors is becoming increasingly widespread in SMC detection areas and will play an invaluable role in the future.

In mycotoxin removal, compared with the traditional physical adsorption and chemical degradation methods, mycotoxin degradation enzymes have the advantages of higher catalytic efficiency, better specificity, and greater safety, which are the most promising degradation methods. However, the application of natural enzymes in the removal of mycotoxins is limited by their large molecular weight, complex structure, the difficulty of modification, and sensitivity to the environment. With further understanding of the structure and catalytic mechanism of natural enzymes, researchers found that small peptides, nucleic acid, cyclodextrins, metal nanoparticles, and other materials can mimic the catalysis center of natural enzymes to build artificial enzymes with similar activity. Notably, artificial enzymes constructed based on small peptides can mimic the catalytic activities of oxidoreductases, hydrolases, lyases, etc., and have been applied to the degradation of plasticizers, phenolic pollutants, azo dyes, cellulose, amyloid, etc., which provides a new perspective on the removal of mycotoxins.

Given that small peptides have so many potential possibilities and advantages in mycotoxin removal, we hope more researchers in related fields can participate in the discovery of more small peptides with a mycotoxin degradation function and investigate the principle and conformational relationship of the peptide-based mycotoxin removal of artificial enzymes to open up a new way for mycotoxin degradation. Meanwhile, owing to the excellent advantages of small peptides in constructing immunobiosensors, many researchers are already focusing on research into the applications of small peptides in mycotoxin detection, and we should pay more attention to promoting its practical application.

Currently, the major challenge is obtaining small peptide sequences with specific functions quickly and precisely; this requires a deep understanding of the chemical properties, folding patterns, and structures of small peptides. Notably, with the help of peptide structure prediction software such as PEP-FOLD, Alphafold2, and RoseTTAFold, the structure of a specific small-peptide sequence can be more easily and accurately predicted, which facilitates the rational design of small-peptide sequences. The construction of more small peptides with specific functions by rational design will be a future trend. Overall, it will provide new solutions for the detection and removal of mycotoxins or other small-molecule contaminants in the future in a greener, safer, and more efficient way.

## Figures and Tables

**Table 1:** Maximum levels of mycotoxins in agricultural products and foods.

Mycotoxin	Country *	Maximum Level (ML) (ug/kg)	ML in Infant and Young Children’s Food (µg/kg)
AFB_1_	China	5–20	0.5
	EU	AFB_1_: 2–12 AFB_1_ + AFB_2_ + AFG_1_ + AFG_2_: 4–15	0.1
	U.S.	AFB_1_ + AFB_2_ + AFG_1_ + AFG_2_: 20	-
	CAC	AFB_1_ + AFB_2_ + AFG_1_ + AFG_2_: 10–15	-
AFM_1_	China	0.5	0.5
	EU	0.05	0.025
	U.S.	0.5	-
	CAC	0.5	-
OTA	China	2–10	-
	EU	2–80	0.5
	CAC	5	-
ZEN	China	60	-
	EU	50–400	20
DON	China	1000	-
	EU	500–1750	200
	U.S.	1000	-
	CAC	1000–2000	200
PAT	China	50	-
	EU	25–50	10
	U.S.	50	-
	CAC	50	-
FMs	EU	FB_1_ + FB_2_: 800–4000	FB_1_ + FB_2_: 200
	U.S.	FB_1_ + FB_2_ + FB_3_: 2000–4000	-
	CAC	FB_1_ + FB_2_: 2000–4000	-
CIT	EU	2000	-

* Data obtained from: (1) The People’s Republic of China National Standard GB2761-2017; (2) European Union (EU): Regulations (EC) Nos. 2002/32/EC, 1881/2006, 2021/1399); (3) The United States (U.S.) Food and Drug Administration: Mycotoxins: toxins found in food infected by certain molds or fungi (Search date 2022.9.28); (4) Codex Alimentarius Commission (CAC): General standard for contaminants and toxins in food and feed cxs 193-1995 (Amended in 2019). AFB_1_: Aflatoxin B_1_; AFB_2:_ Aflatoxin B_2_; AFG_1_: Aflatoxin G_1_; AFG_2_: Aflatoxin G_2_; FB_1_: Fumonisin B_1_; FB_2_: Fumonisin B_2_; FB_3_: Fumonisin B_3_.

**Table 2:** Small peptides in mycotoxin detection.

Peptide Sequence	Peptide Screening Approach	Detection Principle	Peptide Function	Analyte	IC_50_/SC_50_	Detection Limit	Linear Range	Reference
VYMNRKYYKCCK	Rational design	Peptide-based competitive ELISA	Coating antigen	OTA	3.2 μg/L	1.25 μg/L	1.25–10 μg/L	[48]
IRPMVDP	M13 Ph.D.-7	ELISA	Competing antigen	OTA	-	150 pg/mL	200–8000 pg/mL	[56]
AETYGFQLHAMK	M13 Ph.D.-12	Phage Chemiluminescent ELISA	Competing antigen	OTA	0.04 ng/mL	0.005 ng/mL	0.006–0.245 ng/mL	[57]
AEDRPFQLHLPV	M13 Ph.D.-12	CLEIA	Coating antigen	OTA	0.82 ng/mL	-	0.31–2.17 ng/mL	[58]
AEDRPFQLHLPV	M13 Ph.D.-12	Dot immunoassay	Coating antigen	OTA	-	5.0 ng/mL	-	[58]
Biotin-KSGGGSNLHPK	Rational design	Dot-blot assay	Recognition element	OTA	-	0.49 μg/kg	-	[59]
GMVQTIFGGGSK-Biotin	M13 Ph.D.-7	Phage-free peptide ELISA	Competing antigen	OTA	0.024 ng/mL	0.001 ng/mL	0.005–0.2 ng/mL	[60]
DLLWVPST	Phage display random linear 8-mer peptide library	Nc-MCLEIA	Anti-immune complex peptide	AFB_1_	0.089 ng/mL	0.006 ng/mL	0.019–0.407 ng/mL	[61]
YSWHEWYIPQLS	M13 Ph.D.-12	MB-dcELISA	Competing antigen	AFB_1_	0.75 ng/mL	0.13 ng/mL	0.24–2.21 ng/mL	[62]
CVPSKPGLC	M13 Ph.D.-C7C	Bp-ELISA	Competing antigen	AFB_1_	0.92 ng/mL	0.09 ng/mL	0.23–3.36 ng/mL	[63]
CNVLPFDSIFRF	Rational design	Electrochemical immunosensor	Recognition element	AFB_1_	-	9.4 × 10^−4^ mg/L	0.01–20 μg/L	[64]
ACPYPNHPYC	M13 Ph.D.-12	Electrochemical immunosensor	Competing antigen	DON	58.26 ng/mL	0.07 g/mL	0.1–10,000 pg/mL	[65]
AIRMIRIRTS	M13 Ph.D.-12	D_8_-MBP ELISA	Coating antigen	DON	57.98 ng/mL	9.83 ng/mL	11.32–286.77 ng/mL	[66]
ESYWATVPWTRH	M13 Ph.D.-12	Phage-based dot-immunoassay	Coating antigen	ZEN	1.8 ng/mL	50 mg/kg	-	[67]
DAVILLM	M13 Ph.D.-7	ELISA	Competing antigen	ZEN	-	100 pg/mL	100–10,000 pg/mL	[68]
DAVILLM	M13 Ph.D.-7	PEC-ELISA	Competing antigen	ZEN	-	10^−6^ ng/mL	10^−6^–1 ng/mL	[69]
DAVILLM	M13 Ph.D.-7	MSR-system	Competing antigen	ZEN	-	1.06 × 10^−7^ ng/mL	10^−7^–10^−1^ ng/mL	[70]
CMTTLFGEDC	Phage display random linear 8-mer peptide library	P-MCLEIA	Competing antigen	ZEN	31.4 pg/mL	4.3 pg/mL	0.0086–0.1475 ng/mL	[71]
GWWGPYGEIELL	M13 Ph.D.-12	Bioluminescent ELISA	Competing antigen	ZEN	11.0 ng/mL	4.2 ng/mL	6.2–19.6 ng/mL	[72]
GWWGPYGEELLGGGSK-Biotin	M13 Ph.D.-12	ULISA	Competing antigen	ZEN	0.16 ng/mL	0.02 ng/mL	0.05–0.50 ng/mL	[73]
--	Phage display random linear 12-mer peptide library	P-dcFLISA	Coating antigen	ZEN	0.301 ng/ml	0.023 ng/mL	0.060–1.531 ng/ml	[74]
ACWELPTLACGGGS	M13 Ph.D.-C7C	ELISA	Coating antigen	FB_1_	6.06 ng/mL	1.18 ng/mL	1.77–20.73 ng/mL	[75]
NNAAMYSEMATD	M13 Ph.D.-12	ELISA	Coating antigen	FB_1_	2.15 ng/mL	0.32 ng/mL	-	[76]
TTLQMRSEMADD	M13 Ph.D.-12	ELISA	Coating antigen	FB_1_	1.26 ng/mL	0.21 ng/mL	-	[76]
VTPNDDTFDPFR	M13 Ph.D.-12	ELISA	Competing antigen	FB_1_	37.1 ng/mL	11.1 ng/mL	0.13–25.6 ng/mL	[77]
VTPNDDTFDPFRGGGSK-Biotin	M13 Ph.D.-12	MB-ELISA	Competing antigen	FB_1_	1.85 ng/mL	0.029 ng/mL	0.13–25.6 ng/mL	[78]
WLTPVGELV	Phage display random cyclic 8-, 9-, 10-mer peptide libraries	Competitive P-ELISA	Competing antigen	Clothianidin	3.83 ng/mL	1.11 ng/mL	1.11–13.20 ng/mL	[79]
AVFTDQWWTG	Phage display random cyclic 8-, 9-, 10-mer peptide libraries	Noncompetitive P-ELISA	Anti-immune complex peptide	Clothianidin	0.45 ng/mL	0.26 ng/mL	0.26–0.76 ng/mL	[79]
CTMHLSVYC	M13 Ph.D.-C7C	ELISA	Anti-immune complex peptide	Ethyl carbamate	1.66 ng/mL	0.54 ng/mL	0.87–3.20 ng/mL	[80]
((CSGLAEFMSC)_2_K)_2_KK-FITC	Rational design	Competitive FPIAs	Competing antigens	Benzothiostrobin	19.71 ng/mL	4.27 ng/mL	4.27–129.42 ng/mL	[81]
((CPDIWPTAWC)_2_K)_2_KK-FITC	Rational design	Noncompetitive FPIAs	Anti-immune complex peptide	Benzothiostrobin	40.43 ng/mL	9.27 ng/mL	9.27–210.62 ng/mL	[81]
CFNGKDWLYC	Phage display random cyclic 8-mer peptide libraries	PHAIA	Coating antigen	PBA	0.31 ng/mL	0.05 ng/mL	0.05–2.0 ng/mL	[82]

OTA: Ochratoxin A; AFB_1_: Aflatoxin B_1_; DON: Deoxynivalenol; ZEN: Zearalenone; FB_1_: Fumonisin B_1_; PBA: Phenoxybenzoic acid.

**Table 3:** Types and catalytic activities of some peptide-based artificial enzymes.

Peptide-Based Artificial Enzyme	Structure	Substrate	Reaction Condition	*k*_cat_ (×10^−3^ s^−1^)	*K*_M_ (mM)	*K*_cat_/*K*_M_ (M^−1^ s^−1^)	References
Peptide-based oxidoreductase mimics							
F-Cu(II)	Nanosheets	2,4-DP	PBS buffer (pH 7.4), 22 °C	11.90 × 10^3^	0.19	62.65	[97]
H15	Nanosheets or Ribbons	TMB	50 mM MES (pH 7.0), 25 °C	0.13	-	0.70	[101]
		H_2_O_2_		0.11	-	3.13 × 10^−2^	
Hemin(Phe+His)	Nanofibers	Pyrogallol	Toluene	17.42 × 10^3^	-	-	[102]
			Phosphate buffer (10 mM, pH 7.4)	0.82 × 10^3^			
Gel-6	Nanofibers	Pyrogallol	Toluene	25.95 × 10^3^	-	-	[103]
			Phosphate buffer (10 mM, pH 7.4)	1.51 × 10^3^			
His-C7-Mn(II)	Nanospheres	OPD	pH 7.1, 25 °C, 10 mM OPD	0.13 × 10^3^	8.45	15.38	[104]
		H_2_O_2_	pH 7.1, 25 °C, 280 mM H2O2	0.48 × 10^3^	53.63	8.94	
NapFFH	Nanotubes	*p*NPA	PBS buffer (10 mM, pH 7.4), 25 °C	1.96	0.93	2.11	[105]
NapFFHCu	Shorter nanotubes	TMB	PBS buffer (10 mM, pH 7.4), 25 °C	0.34	0.77	0.44	[105]
		H_2_O_2_		0.26	7.77	0.03	
NapFFHH	Nanotubes	*p*NPA	PBS buffer (10 mM, pH 7.4), 25 °C	0.37	2.56	0.14	[105]
NapFFHHCu	Shorter nanotubes	TMB	PBS buffer (10 mM, pH 7.4), 25 °C	0.11	4.32	0.26	[105]
		H_2_O_2_		0.39	4.84	0.08	
NapFFRH	Nanotubes	*p*NPA	PBS buffer (10 mM, pH 7.4), 25 °C	1.37	5.68	0.24	[105]
NapFFRHCu	Thinner nanotubes	TMB	PBS buffer (10 mM, pH 7.4), 25 °C	0.54	0.40	1.35	[105]
		H_2_O_2_		0.95	2.32	0.41	
NapFFKH	Nanotubes	*p*NPA	PBS buffer (10 mM, pH 7.4), 25 °C	1.13	4.19	0.27	[105]
NapFFKHCu	Thinner nanotubes	TMB	PBS buffer (10 mM, pH 7.4), 25 °C	2.67	1.28	2.08	[105]
		H_2_O_2_		1.74	1.11	1.57	
NapFFDH	Nanotubes	*p*NPA	PBS buffer (10 mM, pH 7.4), 25 °C	1.24	5.32	0.23	[105]
NapFFDHCu	Nanospheres	TMB	PBS buffer (10 mM, pH 7.4), 25 °C	0.28	1.90	0.15	[105]
		H_2_O_2_		0.20	7.61	0.03	
NapFFSH	Nanotubes	*p*NPA	PBS buffer (10 mM, pH 7.4), 25 °C	1.01	8.80	0.12	[105]
NapFFSHCu	Nanospheres	TMB	PBS buffer (10 mM, pH 7.4), 25 °C	0.51	0.79	0.64	[105]
		H_2_O_2_		1.12	16.22	0.07	
LMLHLFL-hemin	Nanofibers	TMB	Phosphate buffer (100 mM, pH 6)	0.47 × 10^3^	9.9 × 10^−3^	0.47 × 10^5^	[106]
		ABTS		2.38 × 10^3^	7.8 × 10^−3^	3.06 × 10^5^	
DhHP-6	-	TMB	PBS buffer (10 mM, pH 7.4), 30 °C	-	0.55	Vm = 20.68 × 10^−8^ M s^−1^	[96]
		H_2_O_2_			0.47	Vm = 3.89 × 10^−8^ M s^−1^	
DhHP-6-c-ZrMOF	Nanoparticles	TMB	PBS buffer (10 mM, pH 7.4), 30 °C	-	0.37	Vm = 35.24 × 10^−8^ M s^−1^	[96]
		H_2_O_2_			0.35	Vm = 5.484 × 10^−8^ M s^−1^	
Peptide-based hydrolase mimics							
CAII	Carbonic anhydrase (PDB:2CBA)	*p*NPA	pH 7.0, 25 °C	37	22.1	1670	[107]
			pH 7.5, 25 °C	-	30.53	2600	
			pH 8.0, 25 °C	53	20.7	2550	
			pH 9.0, 25 °C	56	23.9	2320	
E3H15	α-helix	*p*NPA	Tris-HCl (50 mM, pH 7.3), 25 °C	0.19	0.98	0.20	[108]
E3H15/K3				0.23	1.49	0.15	
Au@E3H15	Gold nanoparticles			0.72	0.31	2.18	
Azo-GFGH	Nanofibers	*p*NPA	PBS (50 mM, pH 7.4), 25 °C (before UV)	3.67	15.56	0.23	[109]
			PBS (50 mM, pH 7.4), 25 °C (after UV)	3.00	15.97	0.18	
1NF	Nanofibers	*p*NPA	HEPES (10 mM, pH 7.4), 25 °C	1.28	1.22	1.00	[110]
1TB	Twisted bundles			1.07	0.63	1.71	
2NF	Nanofibers			1.15	2.01	0.575	
(di-2⊂γ-CD)NS	Nanosheets			0.54	3.14	0.172	
Im-KL	Nanotubes	p-nitrophenyl 4-oxopentanoate 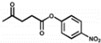	HEPES (25 mM, pH 8)	1.50	0.74	2.1	[111]
				1.17	2.4	0.48	
Im-RL		p-nitrophenyl pentanoate 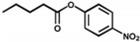		1.17	0.32	3.6	
				0.50	0.3	1.8	
MAX1-H2H5	β-structured fibrils	*p*NPA	Tris (15 mM, pH 7.4), 25 °C	2.10	2.99	0.71	[112]
			Tris (15 mM, pH 9.0), 25 °C	37.2	3.07	12.1	
			CAPS (15 mM, pH 10.0), 25 °C	9.98	0.41	24.3	
		*p*NPMA	Tris (15 mM, pH 9.0), 25 °C	69.8	0.15	452	
		*p*NPP	Tris (15 mM, pH 9.0), 25 °C	7.59	0.13	58.1	
MAX1-H2S5	β-structured fibrils	*p*NPA	Tris (15 mM, pH 7.4), 25 °C	0.78	0.75	1.04	[112]
			Tris (15 mM, pH 9.0), 25 °C	17.4	1.31	13.3	
			CAPS (15 mM, pH 10.0), 25 °C	47.4	0.48	99.4	
		*p*NPMA	Tris (15 mM, pH 9.0), 25 °C	38.9	0.19	203	
		*p*NPP	Tris (15 mM, pH 9.0), 25 °C	7.27	0.05	145	
CoA-HSDmax	Nanofibers	*p*NPA	HEPES (10 mM, pH7.5), 35 °C	3.00	16.29	0.19	[113]
SA-H				2.00	20.03	0.096	
Q11H	Nanofibers	*p*NPA	PBS (10 mM, pH 7.4)	1.95	21.68	0.09	[114]
Q11HRmax				2.64	17.63	0.15	
SA-H	Nanofibers	*p*NPA	PBS (10 mM, pH 8.0), 25 °C	6.73 × 10^−2^	7.79	0.088	[93]
NIP-H				5.93 × 10^−2^	4.28	0.14	
AMIP-H				6.87 × 10^−2^	2.66	0.258	
IHQ-NP	β-sheets	*p*NPA	Tris-HCl (50 mM, pH8.0), 25 °C	4.49	1.61	2.79	[115]
Zn(II)-IHQ-NP				10.53	1.21	8.69	
Au@IHQ-NP	Nanoparticles			2.41	0.49	4.95	
Zn(II)-Au@IHQ-NP				7.97	0.50	16.06	
F–Zn(II)	Amyloid-like structure	*p*NPA	Deionized water (pH 7.0), 22 °C	-	-	76.54	[116]
			Tris-HCl (25 mM, pH 7.0), 22 °C	-	-	10.62	
VK2H	Nanofibers	*p*NPA	Tris-HCl (15 mM, pH 9.0)	70	3.65	19.18	[117]
Ac-IHIHIYI-NH_2_	Nanofibers	*p*NPA	Tris-HCl (25 mM, pH 8.0), 37 °C	8.26 × 10^3^	0.02	355	[118]
HKH-LLLAAA(K)-palmitoyl	Nanofibers	DNPA	HEPES(50 mM, pH 7.4), 25 °C	16.7	0.85	19.76	[119]
PepNTs-His-Argmax	Nanotubes	*p*NPA	HEPES(10 mM, pH 7.5), 25 °C	1.38	0.76	1.82	[120]
MID1-zinc	Artificial metalloprotease (PDB:3V1C)	*p*NPA	HEPES (40 mM, pH 7.0), 25 °C	42	1.18	35	[121]
			HEPES (40 mM, pH 7.5), 25 °C	81	0.90	90	
			HEPES (40 mM, pH 8.0), 25 °C	1.5 × 10^2^	0.82	190	
			HEPES (40 mM, pH 8.5), 25 °C	2.2 × 10^2^	0.47	470	
			HEPES (40 mM, pH 9.0), 25 °C	2.8 × 10^2^	0.42	660	
		pNPP	HEPES (40 mM, pH 8.5), 37 °C	0.2	1.2 × 10^−2^	14	
AuNPs@POMD-8pep	Nanoparticles	BAPNA	Tris-HCl 7.4, 37 °C	2.18 × 10^6^	0.16 g·L^–1^	8.26 × 10^5^ L·g^−1^·min^−1^	[122]
JAL-AK22	Small peptide	MMP18-33	PBS (pH 7.4), 37 ◦C	-	0.17	-	[123]
		MMP18-40			0.15		
JAL-TA9	Small peptide	Aβ1-20	PBS (pH 7.4), 37 ◦C	-	1.27	-	[123,124,125]
		Aβ11-29			0.56		
		MMP18-33			0.17		[126]
		MMP18-40			0.15		
ANA-TA9	Small peptide	Aβ11-29	PBS (pH 7.4), 37 ◦C	0.02	0.32	-	[100,127,128]
ANA-SA5	Small peptide	Aβ11-29	PBS (pH 7.4), 37 ◦C	0.79 × 10^−2^	0.13	-	[127]
ANA-YA4				0.01	0.15		
Peptide-based lyase mimics							
F-Zn(II)	Needle-like architectures	*p*NPA	Deionized water (pH 7.0), 22 °C	-	0.17	76.54	[116]
		CO_2_ + H_2_O	Tris buffer (20 mM, pH 8)	7.80×10^3^	8.10	962.00	
YLK-18-opt	-	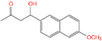	5% CH_3_CN, 42.5 mM Na phosphate (pH 7.5), 25 °C	3.50 × 10^−3^	1.80	*k*_cat_/*k*_uncat_ = 540	[129]
		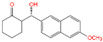	5% DMSO, 42.5 mM Na phosphate (pH 7.5), 25 °C	6.83 × 10^−3^	0.90	*k*_cat_/*k*_uncat_ = 170	
FT-YLK-3		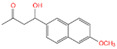	5% CH_3_CN, 42.5 mM Na phosphate (pH 7.5), 25 °C	9.33 × 10^−3^	1.80	*k*_cat_/*k*_uncat_ = 1400	
		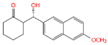	5% DMSO, 42.5 mM Na phosphate (pH 7.5), 25 °C	2.00×10^−3^	1.1	*k*_cat_/*k*_uncat_ = 500	
FluS303-FTYLK3	-	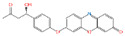	10% CH_3_CN, 42.5 mM Na phosphate (pH 7.5), 25 °C	3.83 × 10^−3^	8.00 × 10^−3^	4.83 × 10^−1^	[130]
		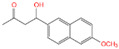	5% CH_3_CN, 42.5 mM Na phosphate (pH 7.5), 25 °C	12.30 × 10^−3^	1.10	1.12 × 10^−2^	
FT-YLK-3		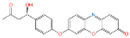	10% CH_3_CN, 42.5 mM Na phosphate (pH 7.5), 25 °C	3.33 × 10^−3^	0.13	2.50 × 10^−2^	
		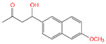	5% CH_3_CN, 42.5 mM Na phosphate (pH 7.5), 25 °C	9.33 × 10^−3^	1.80	5.17 × 10^−3^	
Ac-IHIHIQI-NH_2_	Nanofibers	*p*NPA	Tris (25 mM, pH 8), 22 °C	26	0.4	62	[131]
[Hg(II)]S[Zn(II)(H2O/OH-)]N(TRIL9CL23H)3n+	Artificial metalloprotease (PDB:3PBJ)	*p*NPA	HEPES(50 mM, pH 7.5), 25 °C	2.2	1.6	1.38	[107]
			HEPES(50 mM, pH 8.0), 25 °C	5.4	1.7	3.1	
			CHES (50 mM, pH 8.5), 25 °C	12	1.9	6.0	
			CHES (50 mM, pH 8.75), 25 °C	21	2.0	10.8	
			CHES (50 mM, pH 9.0), 25 °C	38	2.1	17.6	
			CHES (50 mM, pH 9.5), 25 °C	40	1.7	23.3	
